# Expanding the Landscape of Chromatin Modification (CM)-Related Functional Domains and Genes in Human

**DOI:** 10.1371/journal.pone.0014122

**Published:** 2010-11-29

**Authors:** Shuye Pu, Andrei L. Turinsky, James Vlasblom, Tuan On, Xuejian Xiong, Andrew Emili, Zhaolei Zhang, Jack Greenblatt, John Parkinson, Shoshana J. Wodak

**Affiliations:** 1 Program in Molecular Structure & Function, Hospital for Sick Children, Toronto, Canada; 2 Department of Biochemistry, University of Toronto, Toronto, Canada; 3 Department of Molecular Genetics, University of Toronto, Toronto, Canada; 4 Terrence Donnelly Centre for Cellular and Biomolecular Research, Toronto, Canada; 5 Banting and Best Department of Medical Research, Toronto, Canada; Wellcome Trust Sanger Institute, United Kingdom

## Abstract

Chromatin modification (CM) plays a key role in regulating transcription, DNA replication, repair and recombination. However, our knowledge of these processes in humans remains very limited. Here we use computational approaches to study proteins and functional domains involved in CM in humans. We analyze the abundance and the pair-wise domain-domain co-occurrences of 25 well-documented CM domains in 5 model organisms: yeast, worm, fly, mouse and human. Results show that domains involved in histone methylation, DNA methylation, and histone variants are remarkably expanded in metazoan, reflecting the increased demand for cell type-specific gene regulation. We find that CM domains tend to co-occur with a limited number of partner domains and are hence not promiscuous. This property is exploited to identify 47 potentially novel CM domains, including 24 DNA-binding domains, whose role in CM has received little attention so far. Lastly, we use a consensus Machine Learning approach to predict 379 novel CM genes (coding for 329 proteins) in humans based on domain compositions. Several of these predictions are supported by very recent experimental studies and others are slated for experimental verification. Identification of novel CM genes and domains in humans will aid our understanding of fundamental epigenetic processes that are important for stem cell differentiation and cancer biology. Information on all the candidate CM domains and genes reported here is publicly available.

## Introduction

Chromatin modification (CM) encompasses chromatin remodeling (eviction, deposition, or sliding of nucleosomes along DNA), histone exchange (substitution of core histones with histone variants) and covalent modification of DNA (methylation) and histones (acetylation, methylation, ubiquitylation, phosphorylation, etc.). By altering chromatin structure globally (e.g., chromatin condensation and heterochromatin formation) and locally (e.g., mobilization of nucleosomes), CM dictates access to DNA, thereby playing vital roles in the regulation of all DNA-templated processes, such as transcription and DNA recombination, replication, and repair [1]. For instance, post-translational modifications of histones, one of the many forms of CM, are crucial for the regulation of gene activity. Specifically, histone hyperacetylation is positively correlated with actively transcribed genes [2]. Tri-methylation of H3K4 (H3K4me3) or H3K36 (H3K36me3) is associated with gene activation [1], while H3K9me3 or H3K27me3 is associated with transcriptional repression and heterochromatin formation [3]. In embryonic stem cells, co-existence of both H3K4me3 and H3K27me3 in promoter regions marks key developmental genes that are in poised states [4], [5].

**Figure 1:**
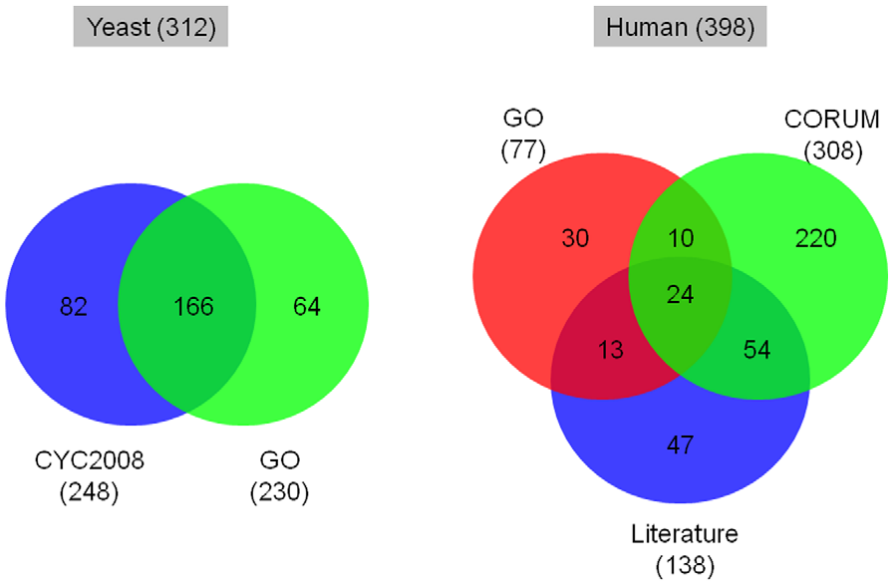
Venn diagrams illustrating the overlap between experimentally characterized CM genes from various data sources in yeast and human. Numbers in parentheses denote the number of genes. Refer to the text for the detailed sources of the genes in each set.

CM has been most extensively studied in the budding yeast, a simple unicellular eukaryote that is amenable to experimental manipulations. However, our knowledge of these processes in human remains very limited. This situation is illustrated by the paucity of genes annotated as CM-related for human as compared to the yeast in the Gene Ontology (GO) database [6]. For yeast, 230 genes are associated with CM in the GO hierarchy on the basis of direct experimental evidence. The corresponding number of human genes is only 77 (Figure 1). Including yeast and human genes coding for components of CM-related protein complexes [7], [8] and other genes derived by curating the recent literature (See Materials and Methods for detail), expands the list of yeast CM genes to a total of 312 members, whereas the expanded list of human CM genes does not exceed 398. This latter number is comparatively small, considering the ∼3-fold larger size of the human genome and the existence in human of more than 200 distinct cell types. Hence, many more CM-related genes remain to be discovered in humans.

In this study we use computational methods to identify new proteins (genes) and domains within proteins, with CM-related function in the human genome. These methods exploit information on protein domains, both CM-related and others, currently annotated by the Pfam database [9].

Substantial evidence has been accumulated recently that chromatin modifying factors exhibit distinct protein domains that perform specific functions, such as the SET domain (a catalytic domain of many histone lysine methyltransferases), Bromodomain (responsible for recognition of acetylated histone lysine) and Chromodomain (responsible for binding of methylated histone lysine) [10], [11]. In fact, the majority of eukaryotic proteins contain domains that carry out specific functions (not necessarily CM related) and have independent evolutionary histories [12], [13]. The different types of domains in a protein - its domain composition - and even more so, the arrangements of these domains along the polypeptide sequence, - the domain architecture - reveal a great deal about the protein's functions [14], [15]. Even a simple measure such as domain composition has been very informative in this regard. Proteins with the same domain composition are often evolutionarily related and have the same or similar functions [12]. This observation has been exploited to predict protein function [16], cellular localization [17] and protein-protein interactions [18], [19].

Here, we analyze respectively, the abundance and the pair-wise domain-domain co-occurrences of 25 domains found in well-documented CM proteins in 5 model organisms: the yeast *Saccharomyces cerevisiae*, the worm *Caenorhabditis elegans*, the fly *Drosophila melanogaster*, the mouse *Mus musculus* and the human *Homo sapiens*. This analysis allows us to evaluate the relative enrichment of known CM domain families between different organisms and to estimate the promiscuity of CM domains, or the degree to which they tend to co-occur with different domains across proteins in a genome. Furthermore, computing the propensity of domains to co-occur with known CM domains enables us to annotate 47 additional domains with CM-related functions.

In the final part of our study, we use a consensus Machine Learning approach based on the popular support vector machine (SVM) technique [20], to identify 379 novel candidate CM genes in human on the basis of the domain compositions of known CM genes in yeast and human.

We show that our domain-based analysis produces richer, and by and large complementary, information to function predictions based on orthology relationships [21], and that it yields new insights on how domains contribute to building complexity in CM function in higher eukaryotes.

## Materials and Methods

### Data on genes and domains

Protein-coding genes from yeast, worm, fly, mouse and human, as well as their Pfam A domain compositions [9], were obtained from the Ensembl database [22] version 53 using the BioMart web search tools (December 19, 2008). Pfam B domains were not considered. The number of genes and unique Pfam domains in each of the 5 model organisms is summarized in the Supplementary Table S1.

Function annotations [6] for yeast and human genes were downloaded from the Gene Ontology (GO) databse [6] on December 8, 2008. Yeast protein complexes were retrieved from CYC2008, an updated archive of Curated Yeast Complexes [7]. Human protein complexes were obtained from the CORUM (Comprehensive Resource of Mammalian protein complexes) database [8] on January 19, 2009.

### Datasets of experimentally verified CM genes

#### CM genes in S. cerevisiae

A list of 230 *S. cerevisiae* genes annotated with “chromatin modification” or its child terms was obtained from the Gene Ontology database [6]. We consider “chromatin silencing” as a child term of “chromatin modification” even though they were not linked in the GO hierarchy. This list only included genes with the following GO evidence codes: IDA (inferred from direct assay), IPI (inferred from protein interaction), IGI (inferred from genetic interaction), and IMP (inferred from mutant phenotype).

In addition, 248 genes were retrieved from 60 CM-related complexes that are supported by experimental evidence and archived in the CYC2008 database [7]. The two datasets were combined to yield a consolidated list of 312 experimentally verified yeast CM genes (Figure 1 and Supplementary Table S2).

#### CM genes in human

Experimentally verified human CM genes were obtained from three sources. One is the GO database, from which 217 human genes annotated with ‘Chromatin modification’ or its child terms (see above) were downloaded. Filtering for genes annotated on the basis of experimental evidence only, as described above, reduced the list to 77 genes. In addition, we collected 308 genes from 125 human complexes archived in the CORUM database [8]. These complexes are annotated with the Funcat term ‘DNA conformation modification (e.g. chromatin)’, which is equivalent to the ‘Chromatin modification’ term in the GO [8]. Third, 138 genes encoding chromatin modification factors or histone modifying enzymes were extracted from the recent literature [23], [24]. Figure 1 illustrates the overlap among the genes retrieved from the three sources and the complete list is provided in the Supplementary Table S3.

### Selection of known CM domains

A list of 25 known CM domains was compiled from a survey of the recent literature [3], [10], [11], [23] (Table 1). This list includes 8 catalytic domains of histone modifying enzymes responsible for histone methylation, demethylation, acetylation and deacetylation. It also includes 9 histone modification recognition domains, a catalytic domain of DNA methyltransferases, and 7 DNA-binding domains.

**Table 1:** Selected known CM domains.

Pfam_Acc	Pfam_id	Function
PF00856	SET	Protein lysine methyltransferase activity
PF08123	DOT1	H3K79 methyltransferase activity
PF02373	JmjC	Histone demethylase activity [64], [65], [66]
PF02375	JmjN	Together with JmjC, appears histone demethylase
PF00628	PHD	Methylated or unmethylated histone H3 binding
PF00385	Chromo	Methylated histone H3 binding [67], [68]
PF00567	TUDOR	Methylated histone binding [69], [70], [71]
PF00855	PWWP	H4K20me binding [72]
PF02820	MBT	Methylated histone binding [73], [74]
PF01853	MOZ_SAS	Histone acetyltransferase activity
PF00583	Acetyltransf_1	Acetyltransferase activity, GNAT family
PF00850	Hist_deacetyl	Histone deacetylase activity
PF02146	SIR2	NAD-dependent histone deacetylase activity
PF00439	Bromodomain	Acetylated histone H3, H4 binding [36], [75]
PF03366	YEATS	Putative histone binding domain [43]
PF01426	BAH	H3, H4 tail binding [76], [77]
PF00533	BRCT	Phosphorylated H2A binding [44], [78]
PF00145	DNA_methylase	DNA-binding, DNA methylase activity
PF01429	MBD	Methylated DNA-binding [45]
PF00271	Helicase_C	ATP binding, helicase activity, nucleic acid binding
PF00176	SNF2_N	DNA-binding, ATP binding
PF00249	Myb_DNA-binding	DNA-binding
PF04433	SWIRM	DNA-binding [79], [80]
PF00125	Histone	DNA-binding
PF00538	Linker_histone	DNA-binding

A total of 25 Pfam domains occurring in well-documented CM proteins were selected as known CM domains (See the text for details). Function annotations of domains were obtained from the Pfam database whenever available, or from the literature, otherwise. Numbers in parenthesis denote literature references.

As this study aims at identifying additional CM genes and domains on the basis of known examples, as defined in the Pfam classification, the list of examples was limited to protein domains annotated as most specialized in histone or DNA modification. Hence, many other domains found not only in histone-modifying proteins but also in a large number of proteins involved in other processes, were not considered. Examples of excluded domains are those found in histone modifying kinases, ubiquitin ligases, deiminases, prolyl isomerases and endopeptidases. For instance, we did not utilize *Methyltransf_11* (PF08241), a catalytic domain of methyltransferases, occurring in 28 human proteins, of which only 4 are histone arginine methyltransferase. Other examples of excluded domains are: *zf-C4HC4* (PF00097) found in histone E3 ubiquitin ligase, *PARP* (PF00644) in histone Poly(ADP-ribose) polymerase, and WD40 (PF00400) in some histone tail binding proteins.

### Simulation of pair-wise domain combinations in model organisms

With the goal of identifying statistically significant domain pairs occurring in CM proteins, the following approach was used. For each domain pair (not necessarily adjacent to each other in the protein sequence), we counted the number of proteins in which this pair occurs and empirically estimated the probability of observing this pair by chance. This was done separately for each of the 5 model organisms considered in this study.

Estimation of the background co-occurrence probability was performed using a simulation procedure that involves domain-pair duplication [25]. In applying this procedure to a given genome, domain abundance (the total number of proteins containing a particular domain), the total number of proteins and their size, in terms of the number of distinct domains they contain, were all maintained. Multiple copies of the same domain in a protein were considered as one instance.

Subject to these constraints, domains were randomly shuffled among proteins in a genome following the published procedure [25]. Briefly, at each step, a randomly picked domain was assigned to a randomly picked protein until all domains were assigned to proteins. Whenever a domain pair appeared in a protein, this pair was immediately duplicated, subject to the availabilities of the particular domains and multi-domain proteins in the corresponding genome at any given iteration, and the duplicate pair was assigned to a different randomly chosen multi-domain protein. This random shuffling was performed 10,000 times.

The main role of the duplication step is to improve the correspondence between data on observed domain neighborhood size and domain abundance in genomes, with those derived from the random shuffling procedure [25] (Supplementary Figure S1).

For a pair of distinct domains *i* and *j* (*i≠j*), let A_ij_ be the observed co-occurrence (number of genes containing this pair) in a given genome, and *S_ij_* be the co-occurrence in a randomly shuffled genome. Let *P_ij_* be the fraction of cases satisfying *S_ij_≥A_ij_* in all simulations. For example, if *A_ij_ = 12*, and *S_ij_≥12* occurs in 3 out of 10,000 simulation runs, then *P_ij_ = 3/10000 = 0.0003*. The co-occurrence score (*CS*) for this pair of domains is then defined as:
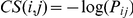
The *CS* score measures how likely it is that *A_ij_* occurs by chance. The greater the *CS* score, the less likely *A_ij_* occurs by chance, and thus the more statistically significant *A_ij_* is.

The same random domain shuffling and duplication model was also used to estimate the level of domain promiscuity. To that end we computed for each domain 1) the size of the actual domain ‘neighborhood’ defined as the number of partner domains with which it co-occurs in different proteins in a given genome (*Ap*), 2) the neighborhood size in the random model, defined as the number of partner domains with which it co-occurs in the simulations (*Sp*), and 3) the empirical probability *P = prob (Sp≤Ap)*, of observing neighborhood sizes smaller than *Ap* in the random model. A low *P* value means that the observed number of partner domains is smaller than in most instances of the simulated genome, indicating in turn that the domain selectively combines with limited number of other domains.

### Identification of candidate CM domains based on co-occurrence

With the goal of using information on domain co-occurrence in order to identify candidate CM domains, we used a graph-based procedure. A weighted graph of domain co-occurrence was constructed for each considered organism. In this graph nodes represent Pfam domains, and two nodes are linked by an edge if they co-occur in a protein, regardless of the distance between them along the protein sequence. Each edge was assigned a weight equal to *CS*, the pair-wise domain co-occurrence score defined above. Furthermore, each graph node was assigned to one of two categories: CM nodes (representing known CM domains) and non-CM nodes (representing non-CM domains).

Given a node *n*, let *N^+^* and *N^−^* represent the sets of CM nodes and non-CM nodes connected to it respectively. An affinity score, *AS*(n), for this node was computed as the fraction of total CS scores associated with CM domain pairs over all domain pairs, counted by linking the node to its first neighbors:
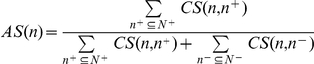
A domain was considered as a candidate CM domain if *AS*(n)>0.5.

### Prediction of CM genes on the basis of their domain composition

Human genes involved in CM processes were predicted on the basis of their domain compositions using a consensus Machine Learning approach based on the Support Vector Machine (SVM) classifier.

The SVM was trained on a reference set composed of positive and negative examples consisting, respectively, of CM genes and non-CM genes.

To have a large enough reference set, we built it from the 20,647 genes in both yeast (*S. cerevisiae*) and human, which contain at least one Pfam domain. CM genes from the other model organisms were not considered because the number of experimentally verified CM genes in these organisms is too small.

The set of positive examples comprised all yeast and human experimentally verified CM genes (see above) that contain at least one Pfam domain, totaling 594 genes (235 from yeast and 359 from human). Defining the set of negative examples (here, non-CM genes from both yeast and human) is a more difficult problem [26]. A common practice is to define such a set as a random sample from the larger set of genes that are currently not among the positive examples [26]. However, this is unsatisfactory because such random samples are likely to be ‘contaminated’ with CM genes yet to be discovered. To correct for this bias, multiple random sampling was combined with a consensus classification strategy, as described below.

#### SVM classification

The domain composition of each gene was represented as a vector ***X_i_*** *= {x_ij_}^d^*, where the dimension *d = 3831*, the total number of unique Pfam domains in human and yeast genomes; and the components *x_ij_ = 1* if gene *i* contains domain *j*, otherwise *x_ij_ = 0*. ***X_i_*** was used as the predictor variables (features) for the SVM classifier.

The SVMs were trained with the SVM_light software [27] using a Gaussian radial basis function kernel (K(***X_i_***, ***X_j_***) = exp(−γ∥***X_i_***−***X_j_***∥^2^) with default C values (trade-off between training error and margin width). The values of γ ( = 0.1) in the above equation and the Cost parameter (which controls the relative weight of training errors on positive examples compared to those on negative examples, and ranges from 3 to 5 depending on the training sample) were determined using a grid-search strategy. The larger Cost values (>2) ensured that training errors on positive examples would outweigh those on the negative examples, as the latter out-numbered the former by a ratio of 17/1.

Since human CM genes are not well annotated, it was not possible to evaluate the performance of the classifier against an independent dataset. We therefore used a Leave-One-Out (LOO) cross validation, widely accepted as a valid performance test in cases where an independent test set is not available [17]. Performance was measured in terms of the Precision, Recall, F-measure and Accuracy, criteria as detailed in Table 2. Not too surprisingly, we see that the performance level is moderate to low (with Precision, Recall and F-measures ranging between 0.54–0.56 for the LOO). We believe that is due to biases in the training dataset. Indeed, owing to the paucity of genes annotated with CM function in human, the negative reference set (non-CM proteins) used for both training and testing is most likely ‘contaminated’ with CM proteins yet to be discovered. This leads to frequent misclassifications, because these contaminant genes will be classified together with known CM genes, and hence be labeled as false positives by the classifier.

**Table 2:** Performance of SVM classifiers.

	Precision	Recall	F-measure	Accuracy
Leave-one-out	0.5424	0.5646	0.5528	0.9489
Re-substitution	0.6539	0.7470	0.6967	0.9636

*Re-substitution* test examines self-consistency of the classification method by classifying on the training set. Precision = TP/(TP+FP), Recall = TP/(TP+FN), F-measure = 2×(Precision×Recall)/(Precision+Recall), Accuracy = (TP+TN)/(TP+FP+TN+FN), where TP = true positive, TN = true negative, FP = false positive, and FN = false negative. The F-measure [81] is the harmonic mean of Precision and Recall, and is a particularly useful performance measure when the dataset is unbalanced such that there are significantly more negative examples than positive ones. We chose not to measure Specificity ( = TN/(TN+FP)) because it is less meaningful in such situations.

False positives may also arise in cases where domains from the same Pfam family are associated with different cellular functions. For instance, some ‘Actin’ domain-containing cytoskeleton proteins that seem to be unrelated to CM are misclassified as CM proteins, most likely due to the fact that ‘Actin’ domains also frequently appear in CM proteins in both yeast and human. In such cases, additional knowledge, or further classification of the domains into sub-families [28], is required to differentiate between CM proteins containing ‘Actin’ domains, and genuine false positives due to “contaminations” coming from the negative training sets.

#### Consensus prediction

To correct for the above-mentioned biases a consensus classification strategy was employed. Genes that are not currently labeled as CM genes were randomly partitioned into two equally sized sets of ‘unknown’ genes. The SVM classifier was then trained using the set of known CM genes as positive examples and one of the ‘unknown’ partitions as negative examples. Genes in the other ‘unknown’ partition were classified using the model obtained with the current training sets. This process was repeated 400 times, with each unknown gene classified approximately 200 times. Only genes classified as CM genes in more than 95% of the repetitions were considered as candidate CM genes. This stringent requirement eliminated 77 genes (17% of all predictions) that are likely to be false positive. For instance, FRAP1, which is a kinase subunit of both mTORC1 and mTORC2 and shares domains with 3 known CM genes, was excluded due to the fact that it was only classified as positive in 93% repetitions although it scored relatively high (0.98) by the SVM classifier.

### Ranking domains based on their relative enrichment in human CM genes

The enrichment of Pfam domains in human CM genes was estimated from the log odds ratio (*LOR*), computed for each domain *d*:
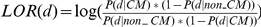
where *P(d|CM)* and *P(d|non-CM)* are the conditional probabilities of observing domain *d* given a CM gene and a non-CM gene, respectively. In cases where *P(d|CM)* or *P(d|non-CM)* equals 0, a background distribution was assumed, taken as 1/(CM+non_CM). *LOR(d)>0* indicates that *d* is relatively enriched in CM genes.

*LOR(d)* was computed for all Pfam domains in CM and non-CM genes in human. The extended set of CM-genes totaled 921 members and comprised the 398 experimentally verified CM genes, 379 candidate CM genes inferred by our SVM procedure, 121 CM genes predicted on the basis of orthology relationships reported in a recent study [21] and 23 annotated electronically by the GO database. The remaining 20495 human genes were taken to represent non-CM genes. All Pfam domains in human were ranked in order of decreasing *LOR(d)* value.

## Results

### Differential expansion of CM domain families across model organisms

**Figure 2:**
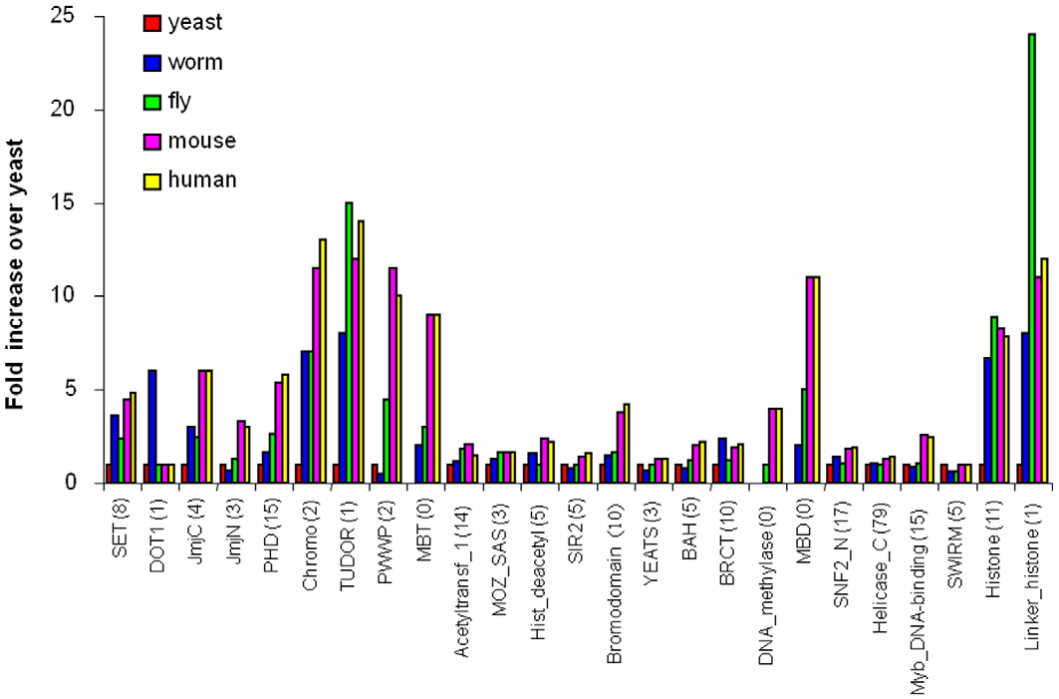
Expansion in the number of known CM domains in 4 model organisms relative to that in yeast. On the X-axis, figures in parentheses following each domain denote the numbers of genes in yeast. Y-axis represents folds of increase over yeast when the number of domain-containing genes is non-zero in yeast, otherwise (for MBT, MBD and DNA_methylase domains), the absolute number of domain-containing genes in each organism.

The fold increase, relative to the yeast *S. cerevisiae*, of the number of genes containing each of the 25 CM-specific domains considered here is plotted in Figure 2 for 4 model organisms (worm, fly, mouse and human). This plot confirms that domains involved in carrying out all basic forms of post-translational covalent histone modifications are conserved from yeast through human and expanded in most cases, generally reflecting the size and complexity of the genomes involved [29], [30], [31], [32]. This reflects the fact that histone modifications, such as lysine acetylation/deacetylation and lysine and arginine methylation/demethylation, carried out in the unicellular budding yeast are conserved in metazoans, from fly to human [23].

Interestingly, our analysis reveals that domains required for histone acetylation (Acetyltransf_1 and MOZ_SAS) and deacetylation (Hist_deactyl and Sir2) are only slightly expanded in the human genome (1.5∼2.2 fold over yeast). On the other hand, the SET family (responsible for all mono-, di- and tri-methylation of various lysine residuals in the N-terminal tail of H3 and H4 (other than H3K79) exhibits a nearly five-fold expansion in human.

A similar trend in differential expansion is observed for domains recognizing histone acetylation marks and methylation marks in agreement with previous observations [33], [34], [35], [36]. Indeed, we find that while the Bromodomain family that binds to acetylated lysine residues is moderately expanded (4 times in comparison to yeast), the variety and the number of protein domains that recognize histone methylation marks are remarkably increased in human (PHD domain: 5 times; the royal family domains (Chromo, TUDOR, PWWP and MBT): 9∼14 times; with the MBT domain absent in yeast). These striking expansions in domains responsible for writing/erasing/reading of histone methylation marks in metazoans likely reflect the greater need for gene repression that is essential for development and tissue-specific gene regulation [37].

Our analysis also reveals that whereas the core histones are well conserved [38], a substantial increase in histone variants and other Histone/Linker_histone domain-containing proteins is evident in higher eukaryotes (7∼12 times over yeast). Particularly noteworthy is the 24-fold increase in the number of Linker_histone domain-containing genes in the fly relative to yeast. Our gene-based analysis may have overestimated the expansion of core histones (H2A, H2B, H3, H4) and linker histones (H1/H5), given that the replication-dependent core histones and linker histones are often encoded by multiple genes [39]. However, there is ample evidence that human and mouse have more than nine types of replication-independent histone variants in comparison to just one (H2A.Z) in yeast [40] and that at least 11 different linker-histone variants exist in mammals [40], [41].

In contrast to these substantial expansions, the catalytic domains of ATP-dependent chromatin remodeling enzymes (SNF2_N, Helicase_C) are well conserved [42] and only moderately expanded. This is also the case for YEATS (a putative histone binding domain) [43], BRCT (a histone phosphorylation mark-recognizing domain) [44], Myb_DNA_binding and SWIRM domains (DNA-binding domains).

An important evolutionary event in chromatin-based processes is the emergence in higher eukaryotes of DNA methylation and related domains (DNA_methylase domain for catalysis and MBD domain for methylated DNA-binding) [45], [46]. We find, indeed, that the number of MBD domain-containing proteins in mouse and human is twice that in fly.

### Co-occurrence of CM domains with other domains

We evaluate the propensity of two domains to co-occur in proteins of a given genome by computing their co-occurrence score CS which ranges from 0 to 9.9. This analysis was performed for all domain pairs where at least one member of the pair was one of the 25 known CM domains considered in this study.

**Figure 3:**
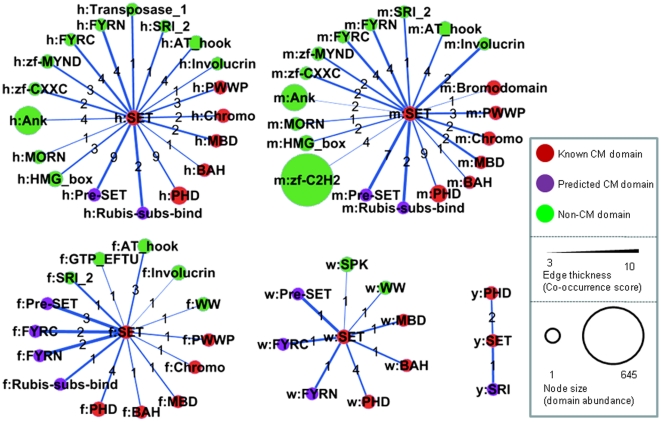
Abundance and combination partners of SET domains in yeast (y), worm (w), fly (f), mouse (m) and human (h) are shown as an illustration of domain neighborhood expansion as a function of domain abundance. See Table 4 for SET domain abundance values in each organism. The prefix in front of each domain name indicates the source organism. Nodes represent domains and links represent co-occurrence relationship in a single protein. Size of the nodes is proportional to the number of domain-containing proteins in each genome, and nodes are colored red, magenta and green to denote known CM domains, candidate CM domains and non-CM domains, respectively. The figures on each edge indicate the numbers of proteins that contain the linked domain pairs. The thickness of edges is proportional to the Co-occurrence Score of the linked domain pairs (See Materials and Methods for definition of Co-occurrence Score).

Results presented in Figure 3 illustrate the propensity of one of the domains, the SET domain, to co-occur with other domains in the 5 model organisms analyzed here. Its highest scoring partner is the Pre-SET domain in all these organisms except in yeast (which lacks the Pre-SET domain). Two other highly scoring partnerships, also conserved from worm to human, are with two DNA- binding domains FYRN and FYRC.

**Figure 4:**
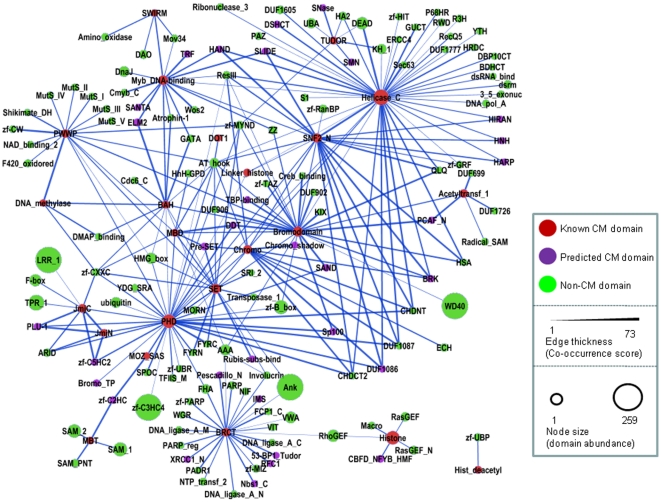
A domain co-occurrence network for known CM domains and their combination partners in human. Nodes represent domains and each link represents co-occurrence relationship of two domains in proteins. Size of the nodes is proportional to the number of domain-containing proteins in each genome, and nodes are colored red, magenta and green, denoting known CM domains, candidate CM domains and non-CM domains, respectively. The thickness of edges is proportional to the Co-occurrence Score for the linked domain pair (See Materials and Methods for definition of Co-occurrence Score).

Figure 4 depicts the domain co-occurrence network involving the CM domains and their co-occurring partner domains in human. Nodes represent individual domains, and the edges, whose thickness is proportional to the CS score, represent co-occurrence relationships in the same genes/proteins. It appears that most CM domains (except for SIR2, Hist_deacetyl and YEATS) tend to co-occur with each other or share a co-occurrence partner with at least one other CM domain. CM domains also co-occur with many non-CM domains. In fact, some non-CM domains exhibit a high co-occurrence score (CS) with several CM domains, suggesting that these non-CM domains may be involved in chromatin modification as well. Equivalent domain co-occurrence networks for 3 other model organisms are presented in the Supplementary Figure S2.

### Prediction of candidate CM domains on the basis of domain co-occurrence

It is reasonable to assume that domains with a high propensity to co-occur with known CM domains may also have CM-related functions. To identify such domains we defined an affinity score (AS), which measures the preference of a domain to co-occur with CM domains relative to non-CM domains. If a domain currently classified as non-CM has a higher affinity for CM domains than for other domains, this domain is inferred to have CM function. Based on this analysis, we identified 47 candidate CM domains (Table 3). Interestingly, we find that 24 of these domains are known or putative DNA/nucleic acid-binding domains (Table 3). Some of them are DNA-binding zinc finger domains (zf-C2H2, zf-CXXC, zf-C2HC, zf-C5HC2 and GATA) that are frequently found in transcription factors. While a few of these DNA-binding domains have been shown to bind specific DNA motifs (ARID, SAND and CBFD_NFYB_HMF), such information is lacking for most of them. Consistent with their role in chromatin modification proposed here, many of these DNA-binding domains appear frequently in chromatin-associated proteins (zf-CXXC, FYRN, FYRC, SAND, CHDCT2, CHDNT, etc.). Another 8 of the 47 candidate CM domains are known or putative protein-protein interaction domains (e.g. ZZ, Nbs1_C, ELM2 and SANTA). The function of 4 candidate CM domains (BRK, Sp100, DUF1086 and DUF1087) is currently unknown.

**Table 3:** Candidate CM domains.

Domain	Molucular Function	Biological Process	Co-occurring CM domain
**Pescadillo_N**	Unkown	cell proliferation	**BRCT**
**IMS**	**damaged DNA binding domain (interpro)**	DNA repair	**BRCT**
**DDT**	**predicted to be a DNA binding domain**	Unkown	**Bromodomain, PHD, MBD**
**PCAF_N**	a domain in the histone acetylase PCAF	regulation of transcription, DNA-dependent	**Bromodomain, Acetyltransf_1**
**Pre-SET**	**structural, stablize SET domain, DNA binding**	chromatin modification	**SET, Chromo, MBD**
**zf-C5HC2**	**predicted to bind DNA**	Unkown	**JmjC, JmjN, PHD**
**Chromo_shadow**	Required for Heterochromatin Spreading.	Unkown	**Chromo**
**PLU-1**	**putative DNA/chromatin binding domain**	Unkown	**JmjC, JmjN, PHD**
**HAND**	**putative DNA/nucleosome binding domain**	ATP-dependent chromatin remodeling	**SNF2_N, Helicase_C, Myb_DNA_binding**
**SNase**	**nucleic acid binding**	Unkown	**TUDOR**
**QLQ**	putative protein interaction domain	regulation of transcription	**SNF2_N, Helicase_C, Bromodomain**
**SLIDE**	**DNA binding**	chromatin remodeling	**SNF2_N, Helicase_C, Myb_DNA_binding**
**NIF**	putative phosphatase	Unkown	**BRCT**
**Rubis-subs-bind**	histone binding	Unkown	**SET**
**RFC1**	ATP binding	DNA replication	**BRCT**
**TBP-binding**	TBP binding to suppress transcription	Unkown	**Bromodomain**
**Bromo_TP**	**predicted to bind DNA**	Unkown	**PHD**
**BRK**	Unkown	Unkown	**SNF2_N, Helicase_C, Bromodomain, Chromo**
**HARP**	Single-strand DNA-depedent ATPase	chromatin modification	**SNF2_N, Helicase_C**
**zf-C2HC**	**DNA binding zinc finger domain**	regulation of transcription, DNA-dependent	**MOZ_SAS, MBT**
**CBFD_NFYB_HMF**	**sequence-specifid DNA binding**	Unkown	**Histone**
**HIRAN**	**predicted to bind DNA, damaged DNA**	Unkown	**SNF2_N, Helicase_C**
**HSA**	**predicted to bind DNA**	Unkown	**SNF2_N, Helicase_C, Bromodomain**
**ELM2**	putative protein interaction domain	Unkown	**BAH, Myb_DNA_binding**
**XRCC1_N**	**specifically binds single-strand break DNA**	single strand break repair	**BRCT**
**TRF**	**Telomeric DNA binding, protein binding**	telomere maintenance via telomerase	**Myb_DNA_binding**
**HNH**	**Nucleic acid binding, endonuclease**	Unkown	**SNF2_N, Helicase_C**
**Nbs1_C**	protein-protein interaction domain	Unkown	**BRCT**
**SANTA**	putative protein-protein interaction domain	Unkown	**Myb_DNA_binding**
**Amino_oxidase**	catalytic domain of LSD1	Unkown	**SWIRM**
**SMN**	RNA binding	mRNA processing, spliceosome assembly	**TUDOR**
**FYRC**	**DNA binding**	Unkown	**PHD, SET**
**DUF1086**	Unkown	Unkown	**SNF2_N, Helicase_C, PHD, Chromo**
**SAND**	**DNA binding**	Unkown	**Bromodomain, PHD**
**DUF1087**	Unkown	Unkown	**SNF2_N, Helicase_C, PHD, Chromo**
**FYRN**	**DNA binding**	Unkown	**PHD, SET**
**ARID**	**DNA binding**	Unkown	**JmjC, JmjN, PHD**
**zf-C2H2**	**DNA binding**	Unkown	**BAH, Myb_DNA_binding, Bromodomain**
**53-BP1_Tudor**	mediates interaction with H3K79me	Unkown	**BRCT**
**Sp100**	Unkown	Unkown	**Bromodomain, PHD**
**CHDNT**	**DNA binding**	regulation of transcription	**SNF2_N, Helicase_C, PHD, Chromo**
**RecQ5**	DNA helicase	Unkown	**Helicase_C**
**zf-CXXC**	**DNA binding**	Unkown	**JmjC, BAH, MBD, SET, PHD, DNA_methylase**
**ZZ**	protein-protein interaction domain	Unkown	**Myb_DNA_binding, Bromodomain**
**CHDCT2**	**DNA binding**	regulation of transcription	**SNF2_N, Helicase_C, PHD, Chromo**
**GATA**	**DNA binding**	regulation of transcription, DNA-dependent	**Myb_DNA_binding, BAH**
**DSHCT**	ATP binding	Unkown	**Helicase_C**

The prediction of candidate CM domains was performed as described in the text. Function annotations are largely based on the literature and Pfam database. *Co-occurring CM domain*: known CM domains that combine with a candidate CM domain in a single protein.

### CM domains are not promiscuous

Domain promiscuity can be defined as a high propensity of a domain to be associated with various domains in different proteins [47]. However, abundant domains are more likely to participate in diverse domain architecture than their less abundant counterparts due to chance events alone [13], [25]. Therefore, to estimate the level of promiscuity of a given domain it is necessary to factor out the influence of domain abundance. This was done here by measuring the extent to which the neighborhood size of a given domain observed in a genome deviates from its neighborhood size in our simulated random model. In both cases, the neighborhood size of a domain is defined as the number of different partner domains with which it co-occurs. More specifically, for each of the 25 known CM domains, we evaluated the empirical probability *P* of observing an equal or smaller neighborhood size by chance (see Methods). A high value of *P* suggests promiscuity, whereas low values indicate selectivity.

The results of this analysis are summarized in Table 4. Dividing the values of *P* into 3 ranges: *P*<0.2 (selective), *P*>0.8 (promiscuous) and 0.2≤*P*≤0.8 (background), we find that 4 of 25 CM domains (DOT1, SIR2, YEATS and Histone) show consistently high selectivity across the 5 model organisms considered here, and another 4 CM domains (Acetyltransf_1, Hist_deacetyl, Helicase_C and Linker_histone) were selective in 4 of the 5 organisms.

**Table 4:** Promiscuity of known CM domains in 5 model organisms.

	Yeast				Worm				Fly				Mouse				Human			
Domain	Ab	Ap	Sp	P	Ab	Ap	Sp	P	Ab	Ap	Sp	P	Ab	Ap	Sp	P	Ab	Ap	Sp	P
SET	8	2	5.2	0.19	29	9	9.5	0.58	19	16	12.6	0.73	36	21	26.2	0.39	39	22	31.9	0.24
DOT1	1	0	0.5	0.00	6	0	1.6	0.00	1	0	0.5	0.00	1	1	0.5	0.12	1	1	0.6	0.13
JmjC	4	4	2.5	0.66	12	7	3.5	0.75	10	11	6.0	**0.83**	24	10	16.7	0.27	24	10	18.8	0.18
JmjN	3	3	1.8	0.55	2	5	0.5	0.27	4	6	2.1	0.65	10	5	6.2	0.46	9	5	6.2	0.45
PHD	15	9	10.3	0.48	25	19	8.0	**0.93**	39	36	28.0	0.78	81	41	60.8	0.17	87	43	71.5	0.06
Chromo	2	3	1.1	0.49	14	10	4.1	**0.83**	14	13	8.9	0.78	23	16	16.0	0.57	26	15	20.6	0.33
TUDOR	NA	NA	NA	NA	8	2	2.2	0.40	15	7	9.7	0.41	12	9	7.8	0.66	14	10	10.3	0.57
PWWP	2	0	1.2	0.00	1	0	0.2	0.00	9	8	5.3	0.73	23	17	15.9	0.62	20	17	15.4	0.64
MBT	NA	NA	NA	NA	2	0	0.5	0.00	3	3	1.5	0.45	9	4	5.6	0.41	9	4	6.2	0.36
MOZ_SAS	3	0	1.8	0.00	4	0	1.0	0.00	5	2	2.7	0.35	5	3	2.8	0.43	5	2	3.3	0.31
Acetyltransf_1	14	3	9.5	0.07	16	4	4.8	0.51	26	5	18.0	0.04	30	5	21.2	0.02	21	5	16.3	0.06
Hist_deacetyl	5	0	3.1	0.00	8	1	2.2	0.22	5	1	2.7	0.19	12	1	7.6	0.04	11	1	7.9	0.04
SIR2	5	1	3.1	0.17	4	0	1.0	0.00	5	0	2.7	0.00	7	0	4.2	0.00	8	0	5.5	0.00
Bromodomain	10	8	6.7	0.70	15	19	4.5	**0.91**	17	24	11.1	**0.96**	38	30	27.5	0.60	42	30	34.4	0.38
YEATS	3	0	1.8	0.00	2	0	0.5	0.00	3	0	1.5	0.00	4	0	2.3	0.00	4	0	2.5	0.00
BAH	5	4	3.2	0.64	4	8	1.0	0.48	6	11	3.3	**0.82**	10	15	6.2	**0.91**	11	14	7.8	**0.87**
BRCT	10	10	6.6	**0.83**	24	7	7.7	0.56	12	14	7.4	**0.87**	19	25	13.1	**0.94**	21	26	16.3	**0.89**
DNA_methylase	NA	NA	NA	NA	NA	NA	NA	NA	1	0	0.5	0.00	4	4	2.2	0.53	4	4	2.5	0.56
MBD	NA	NA	NA	NA	2	4	0.5	0.27	5	9	2.7	0.75	11	9	7.0	0.70	11	9	7.8	0.66
Helicase_C	79	23	55.5	0.00	82	32	30.0	0.56	77	40	56.3	0.15	106	55	79.4	0.14	114	56	92.0	0.05
SNF2_N	17	13	11.7	0.67	24	14	7.6	**0.86**	18	19	11.9	**0.85**	31	24	22.1	0.60	33	24	26.7	0.44
Myb_DNA-binding	15	7	10.3	0.31	13	6	3.8	0.70	16	10	10.5	0.55	38	17	27.6	0.23	37	18	30.3	0.17
SWIRM	5	2	3.1	0.36	3	2	0.7	0.30	3	2	1.5	0.37	5	4	2.9	0.54	5	4	3.3	0.53
Histone	11	1	7.4	0.03	74	0	26.9	0.00	98	5	71.5	0.00	91	6	67.9	0.00	86	6	70.7	0.00
Linker_histone	1	0	0.6	0.00	8	0	2.2	0.00	24	0	16.4	0.00	11	4	6.9	0.33	12	3	8.7	0.14

Promiscuity was estimated using a simulation procedure that allows for domain pair duplication (See the text for details). *Ab*: abundance, defined as the number of proteins containing the domain in a genome. *Ap*: actual number of combination partners of a domain. *Sp*: number of combination partners of a domain obtained in simulations. *P*: empirical probability of observing at most Ap combination partners during simulation of random combinations. A low P value indicates that a domain's actual combination partners are fewer than the results of most random simulations, and indicates that the domain is selective when combining with other domains. For example, in human, the Ap, Sp and P values for the PHD domain are 43, 71.5 and 0.06, respectively; this means that probability P(Sp≤Ap) = 0.06 and, in other words, Ap is less than 94% of simulated Sp values. Conversely, high P value indicates that a domain is promiscuous when combining with other domains. We considered domains with P≤0.2 as selective (marked as underlined in the table) and domains with P>0.8 as promiscuous (marked as bold in the table). Domains with P value in between 0.2 and 0.8 do not deviate from a random combination model. “NA” indicates that the domain is lacking in the organism.

In contrast, no domain was consistently promiscuous across all organisms; BRCT and BAH qualify as promiscuous domains in 4 and 3 organisms, respectively. SNF2_N and Bromodomain were promiscuous in worm and fly only. Interestingly, PHD was promiscuous in worm but selective in mouse and human by the above criteria. The *P* values for the remaining domains did not deviate much from the background level in most of the organisms (Table 4).

These results are in disagreement with those of a recent study in which some CM domains, such as SET, PHD, Chromo, BRCT, JmjC, TUDOR and Bromodomain, were found to be highly promiscuous [48]. This discrepancy is likely due to various factors, including a possible issue with factoring out the effect of domain abundance in the previous study (see Discussion).

### Prediction of CM genes based on domain composition

Using domain composition as a feature, our SVM-based consensus procedure predicted a total of 379 candidate human CM genes (Supplementary Table S4) coding for 329 proteins.

Several lines of evidence lend support to these predictions. First of all, 234 (72%) of the predicted candidate CM genes contain at least one of the 25 known CM domains, even though these domains were not given higher weight than other domains in training the SVM classifiers. Conversely, 19 (76%) of the 25 known CM domains appear in at least one of the candidate CM genes. The most frequently observed CM domains among these genes are: Histone, PHD, Myb_DNA-binding, Acetyltransf_1, Helicase_C, Bromodomain, SFN2_N, SET and Chromo domains (Supplementary Figure S3a). Although the Actin domain is not considered as a CM domain, 26 Actin domain-containing genes were predicted to be candidate CM gene. This is not too surprising given that several chromatin remodeling complexes (Supplementary Table S3) contain components that are annotated with this domain.

Of all our candidate CM genes, 21 are annotated as “chromatin modification” or its child terms with the evidence code IEA (Inferred from Electronical Annotation) in the GO catalog when the annotations were initially downloaded. These genes were excluded from our positive training set because they were not supported by experimental evidence (IEA only). However, among them, JMJD6, a histone arginine demethylase, and TERF2, a telomeric repeat-binding factor, have been re-annotated recently with supporting experimental evidence [49], [50].

Several of our predicted CM genes have also been reported to be involved in CM processes in very recent studies. For example, the jumonji protein (JARID2) was found to form a chromatin modifying complex that methylates H3K9 at the cyclin D1 promoter by recruiting G9a and GLP, two histone methyltransferases [51]. In another example, ALC1 (Amplified in Liver Cancer 1), also known as CHD1L, was recently reported to mediate poly(ADP-ribose)-dependent regulation of DNA repair by binding to poly(ADP-ribosyl)-histone and stimulating nucleosome sliding in an ATP-dependent manner [52].

For completeness, we compared our predictions with those obtained using several publicly available automatic function prediction methods [53], [54], but the results were not very informative, as most of these methods predict molecular or biochemical functions, whereas our study predicts a relatively specific function (“chromatin modification”) in the biological process category.

## Discussion

Chromatin modification and related processes play a key role in gene regulation in eukaryotes. But the molecular players and the complex mechanisms involved remain largely unexplored, particularly in human and other metazoans. Our study produced information that should help advance our knowledge of these processes.

It identified additional proteins and domains in human that are likely to carry out CM-related functions. 18 of these proteins are now subjected to experimental verification in human cells, using the MAPLE technology [55]. The present study also showed that while most CM-domains involved in basic histone modification processes are conserved across 5 model eukaryotes, the number of genes/proteins containing each type of domain tends to increase with the complexity of the organism, in line with the increased spatial and temporal constraints of gene regulation in these organisms.

Prediction of CM genes is a challenging task, in light of the fact that CM is a relatively high level biological process, involving proteins and domains with diverse biochemical activities and molecular functions. Some molecular functions (e.g., histone lysine acetyltransferase activity) of known CM domains are relatively unique to CM, but many others (e.g., protein kinase activity and DNA binding) are shared by a broad variety of different biological processes. This fact compounded with our incomplete knowledge of the ensemble of human CM-genes, contributes to a low performance of our domain composition-based SVM classifier when trained and cross-validated on a single version of the dataset. To circumvent these problems and boost the reliability of the classifier we used a consensus prediction approach, which requires a gene to be classified as CM in multiple classification runs that use different dataset definitions. This more stringent criterion appears to reduce the number of false positives by nearly 20%. We therefore consider the 379 candidate human CM genes (329 proteins) identified by this procedure to represent useful leads for CM function, worthwhile to follow up by experimental analyses.

It is noteworthy that only 110 (30%) of these candidate genes overlap with the list of 231 human genes recently predicted to have CM-related function purely on the basis of orthology relationships [21]. The 269 additional candidates not predicted by orthology were identified here due to the fact that Pfam domain families tend to include more distantly related family members than those identified on the basis of strict orthology relationships. For instance, the orthology-based approach identified 9 Bromodomain-containing human genes as potential CM genes based on their orthology to yeast, worm and fly CM genes [21]. The majority of these genes contain only the Bromodomain. In addition to identifying all these genes, our domain-based approach finds 11 other Bromodomain-containing CM genes, most of which also contain other domains in different arrangements –architectures- along the gene sequence (see Supplementary Table S6). Genes with different domain architectures, cannot be detected as orthologous by the customary reciprocal BLAST criteria [21], [56].

Results of a systematic comparison of our domain-based SVM predictions and those recently derived using orthology relationships [21] are illustrated in Figure S3 of the Supplementary Material. For 121 domains (including 16 known CM domains), we predict more CM genes containing these domains than the orthology-based method (See examples in Figure S3a). However, the latter method outperforms the domain-based approach for 60 other domains, only one of which is a known CM domain. Furthermore, the orthology-based method identifies 20 candidate CM genes that completely lack Pfam domain annotations (Figure S3b). A fraction of these candidate CM genes and others yet to be discovered, may contain novel domain families, or code for proteins with unstructured regions [57]. To identify more distantly related CM-proteins of the latter type by bioinformatics methods, will require methods capable of detecting distantly-related proteins on the basis of sequence information alone [58].

### Domain enrichment in known and predicted human CM genes

A complementary view of the link between Pfam domains and CM-related function can be obtained by estimating the extent to which individual Pfam domains are enriched in human CM genes. To this end we computed the log odds ratio (LOR) of the conditional probabilities of observing a domain, given a CM gene and a non-CM gene respectively (see Methods). This quantity was computed with an extended set of 921 human CM genes, including the 379 genes predicted here by the SVM procedure (See Materials and Methods for the composition of this set).

**Figure 5:**
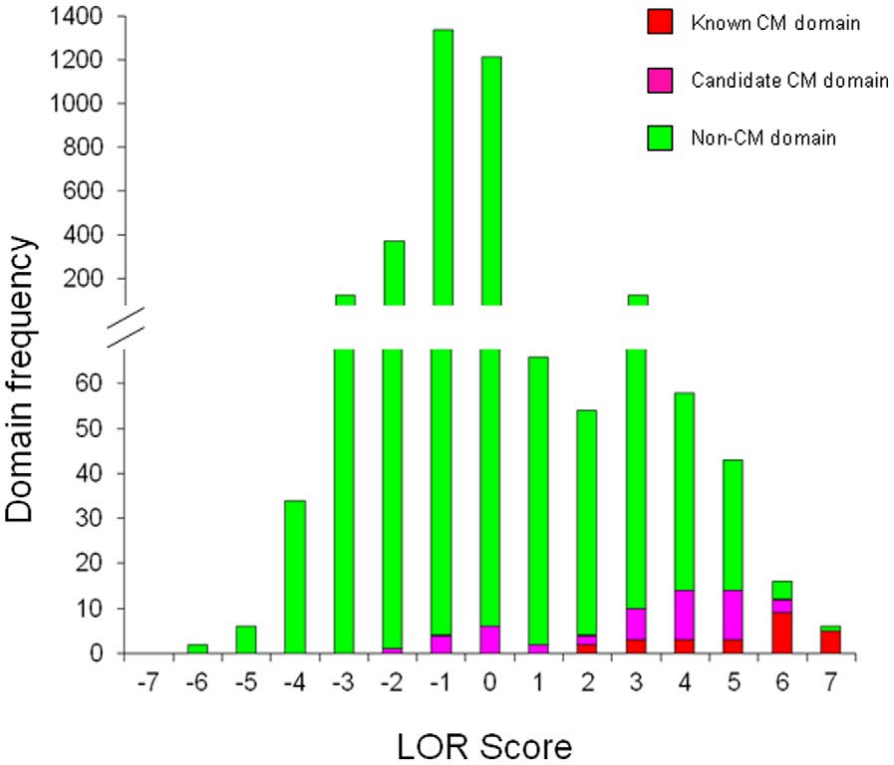
Distribution of log odds ratio (LOR) scores of Pfam domains in the human genome. LOR score measures enrichment of Pfam domains in known and predicted human CM genes. The vast majority of Pfam domains score less than zero, and are thus not enriched or are even under-represented in human CM genes. However, it is clear that the LOR scores of known CM domains and candidate CM domains skew towards the higher end of the LOR spectrum, indicating that these domains are enriched in human CM genes.

Out of the total of 3469 Pfam domains currently annotated in the human genome, only 366 (∼10%) had an LOR above 0 on a scale running from −6.7 to +6.6 and were thus considered as occurring relatively more often in CM genes than non-CM genes (Supplementary Table S5). In fact, 15 (60%) of the 25 domains in our conservative initial set and 26 (55%) of the 47 candidate CM domains identified here on the basis of their propensity to co-occur with members of the initial set occur exclusively in CM proteins. It was rewarding to see that all 25 CM domains in the initial set, as well as 36 of the 47 candidate CM domains, are significantly enriched in CM genes (Figure 5).

Of the remaining 11 domains that are not significantly enriched in CM proteins (LOR<0), all but one (zf-C2H2) do not occur in any of the CM proteins (either experimentally confirmed or predicted) and only co-occur with either of the 3 CM domains, Brct, Tudor or Helicase_C domains. We note however, that the latter 3 domains are least enriched in CM proteins among the 25 domains of our initial set (see Supplementary Table S5), suggesting in turn that they are less specifically related to chromatin modification themselves. Consequently, domains that preferentially associate with them are less reliable CM domain candidates. Conversely, domains, such as Actin, zf-MIZ, MCM, Piwi, MH2, which have high enrichment scores (>5) but are not currently considered as CM-related domains or identified as candidate CM domains by our co-occurrence analysis may actually deserve to be considered as such.

This additional analysis confirms that our methods for predicting CM genes and CM-related domains produce consistent and converging results. These results are also largely consistent with orthology based function predictions, in light of that 25% (231/921) of the CM genes considered in the enrichment calculations were predicted on the basis of orthology.

### Promiscuity of CM domains

An interesting and potentially important finding of our study is that the CM domains have a low propensity to co-occur with many different domains, and are hence rarely promiscuous. CM domains from all five model-organisms analyzed here, including human, display this property, even though we also find that some of these domains are markedly expanded and become increasingly versatile in the process of evolution.

An opposite conclusion has been reached by another recent computational analysis, which reports that CM domains, such as SET, PHD, Chromo, BRCT, JmjC, TUDOR and Bromodomain, are highly promiscuous [48]. A significant factor contributing to this disagreement may be the approach used to normalize the domain co-occurrence rates for domain abundance. The published analysis used an information theoretic approach to measure domain promiscuity. This approach adopted a very liberal threshold for the measure of promiscuity (noted as π in ref 49) so that domain abundance was hardly accounted for. In contrast, our approach involves a random shuffling procedure, which has domain abundance as a built-in constraint and therefore accounts for it naturally. We also verified that it reproduces the power law relationship between domain abundance and domain versatility [13], [25] (Supplementary Figure S1).

The low promiscuity of CM domains uncovered here, seems consistent with the fact that CM proteins mostly operate in the context of multi-protein complexes. These complexes are moreover finely regulated at both the transcriptional and post-transcriptional levels, to afford a high degree of specificity for their targets, and as a result are probably subjected to strong negative selection against promiscuous domain combinations.

### DNA-binding domains in chromatin modification factors

A potentially very significant finding of our study is that CM domains have a propensity to co-occur with DNA-binding domains. Among the 25 known CM domains in our initial set, only 4 (Hist_deactylase, SIR2, YEATS and Acetyltransf_1) do not co-occur with DNA-binding domains. The remaining 21 domains either co-occur with DNA-binding domains in at least one human gene or have DNA-binding activity themselves.

For instance, in the multi-domain protein Sp140, a Bromodomain and a PHD domain co-occur with a SAND domain, which binds a specific DNA motif (TTCG) [59]. In another example, the ARID domain in the JmjC domain-containing histone H3K4 demethylase RBP2 binds to the DNA motif CCGCCC [60]. In the ATP-dependent chromatin remodeling protein ISW2, which contains the DNA-binding domains SNF2 and Helicase_C, two additional DNA-binding domains (HAND and SLIDE domains) are reportedly required to properly anchor and orient the ISW2 complex with respect to the nucleosomes and linker DNA [61].

While histone modification-related domains have been extensively studied recently, the roles of DNA-binding domains in chromatin modification have received little attention so far. For instance, it is not clear what role the DNA-binding ARID domain plays in the JmjC domain-containing histone H3K4 demethylase RBP2 [60].

Sequence-specific DNA-binding domains may recruit histone modifying enzymes or remodeling proteins to the target nucleosomes. Conversely, domains that recognize specific histone modifications may be responsible for directing DNA-binding proteins to their targets loci. A number of different scenarios may be envisaged: 1) if a histone modification-recognizing domains co-occurs with a DNA-binding domain in transcription factors (eg, PHF20), it might serve to link histone modification directly to gene regulation through the concurrent binding to promoter DNA, and histone, thereby enhancing binding specificity; 2) Simultaneous binding of DNA and histone within the same nucleosome may facilitate or impede chromatin remodeling by weakening or strengthening the 14 histone-DNA contacts, respectively; 3) Cooperative binding of DNA and histone in different nucleosomes may lead to the formation of long-range intra- or inter-chromosome chromatin associations [62]. 4) Some of these DNA-binding domains may actually bind non-coding RNAs, which have been recently shown to associate with chromatin modifying complexes in human [63].

Further work is clearly needed to elucidate how chromatin modification and DNA-binding activity are functionally coordinated both spatially and temporally.

## Supporting Information

Figure S1 The relationship between domain abundance and domain neighborhood size in human genome. The logarithm of domain neighborhood size (the number of distinct domains that co-occur with a given domain in different proteins) is plotted against the logarithm of domain abundance (the number of proteins containing the given domain) in human. “log_nabe_human”: the actual data obtained from human genome; “log_sim0_human”: data obtained from simulation experiments in which domains are randomly shuffled among genes in human genome. “log_sim1_human”: data obtained from simulation experiments in which domains are randomly shuffled among genes in human genome, and domain pair duplications were introduced into the simulation procedure to mimic the effects of duplication of multi-domain proteins. Refer to “Material and Methods” in main text for details. A visual inspection indicates that combination of domain pair duplications with random shuffling provides a better approximation of the actual data than random shuffling alone.
(0.20 MB TIF)

Figure S2 Domain co-occurrence network for known CM domains and their combination partners in yeast (a), worm (b), and fly (c). Nodes represent domains and each link represents co-occurrence relationship of two domains in proteins. Size of the nodes is proportional to domain abundance in each genome, and nodes are colored red, magenta and green, denoting known CM domains, candidate CM domains and non-CM domains, respectively. The thickness of edges is proportional to the Co-occurrence Score for the linked domain pair (See Materials and Methods for definition of Co-occurrence Score).
(0.33 MB TIF)

Figure S3 (a) Pfam domains that appear more frequently in our SVM predicted human CM genes than in those predicted by the orthology-based approach. Only the top 36 of 121 such domains are shown. (b) Pfam domains that appear more frequently in CM genes predicted by the orthology-based approach than in those predicted by our SVM-based approach approach. The top 28 of 60 such domains are shown. In both (a) and (b), “Exp_CM”: experimentally verified human CM genes (See SupplementaryTable 3). “svm_prediction”: CM genes predicted by our SVM-based approach only. “orth_prediction”: CM genes predicted by orthology-based approach only. “common_prediction”: CM genes predicted by both approaches. “_Domain_less” on the x-axis of panel (b) denotes CM genes that lack Pfam domain annotations. Note that the orthology-based approach is able to predict CM genes in the absence of Pfam domain annotations, while our SVM-based approach cannot.
(0.19 MB TIF)

Table S1 Number of protein-coding genes and number of unique Pfam-A domains in Yeast, Worm, Fly, Mouse and Human genomes downloaded from Ensembl v.53.
(0.02 MB XLS)

Table S2 List of experimentally verified CM genes in the budding yeast, S. cerevisiae. “-” in the “GO annotation” column and the “CYC2008 complex” column indicates the gene is not annotated with “Chromatin modification” in the Gene Ontology database and not found in any of the CYC2008 complexes, respectively.
(0.08 MB XLS)

Table S3 List of experimentally verified CM genes in human. Again, “-” indicates annotation for the gene is missing. In the “CORUM complex” column, each complex name is followed by a PubMed ID, providing supporting experimental evidence for the complex.
(0.19 MB XLS)

Table S4 List of CM genes predicted with our SVM-based approach in human. “Pfam ID” column provides the name of Pfam domains contained in a gene. The “mean SVM_score” column is the average of SVM scores of predictions (SVM score>0 means the gene is predicted as a CM gene, otherwise, it is predicted as a non-CM gene). The “SVM_std” column is the standard deviation of the mean. The “Frequency of prediction” column indicates how many times the gene is randomly selected for prediction. The value of this column should be around 200, but due to randomization, some genes are picked more frequently than other genes. The “P_value” column provides the probability the SVM score is ≤0, which indicates that gene is classified as a non-CM gene, assuming normal distribution for the SVM scores. This list of 379 genes is divided into two sections. The first section, marked in green, contains genes coding for the 329 non-redundant proteins (including 61 proteins that belong to the actin family and the histone family). The second section, marked in blue, lists genes coding for proteins which have been already identified as known or candidate CM proteins archived in the Supplementary Table 3 or in the first section of this table, respectively.
(0.69 MB XLS)

Table S5 Enrichment of Pfam domains in human CM genes. The “# in CM gene” and the “# in non-CM gene” denote the number of CM genes and non-CM genes containing the particular domain, respectively. LOR: log odds ratio. High LOR indicates the domain is highly enriched in CM genes. The 25 known CM domains and 47 predicted CM domains are highlighted and their LOR values are re-organized in separate sections.
(0.42 MB XLS)

Table S6 Bromodomain-containing CM genes predicted by SVM-based and/or orthology-based approaches.
(0.02 MB XLS)
