# Supplementary figures and images for: Expanding the Landscape of Chromatin Modification (CM)-Related Functional Domains and Genes in Human
Source: PLoS One. 2010 Nov 29;5(11):e14122. doi: 10.1371/journal.pone.0014122 (PMC2993927; doi:10.1371/journal.pone.0014122)

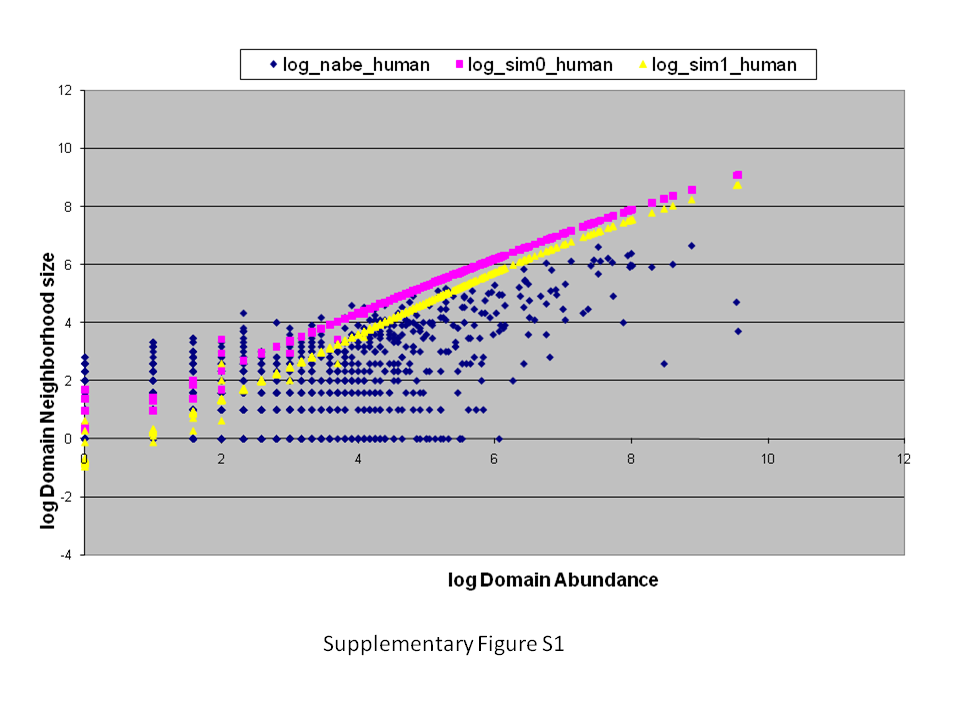

Supplement: Figure S1 — The relationship between domain abundance and domain neighborhood size in human genome. The logarithm of domain neighborhood size (the number of distinct domains that co-occur with a given domain in different proteins) is plotted against the logarithm of domain abundance (the number of proteins containing the given domain) in human. “log_nabe_human”: the actual data obtained from human genome; “log_sim0_human”: data obtained from simulation experiments in which domains are randomly shuffled among genes in human genome. “log_sim1_human”: data obtained from simulation experiments in which domains are randomly shuffled among genes in human genome, and domain pair duplications were introduced into the simulation procedure to mimic the effects of duplication of multi-domain proteins. Refer to “Material and Methods” in main text for details. A visual inspection indicates that combination of domain pair duplications with random shuffling provides a better approximation of the actual data than random shuffling alone. (0.20 MB TIF) [file pone.0014122.s001.tif]

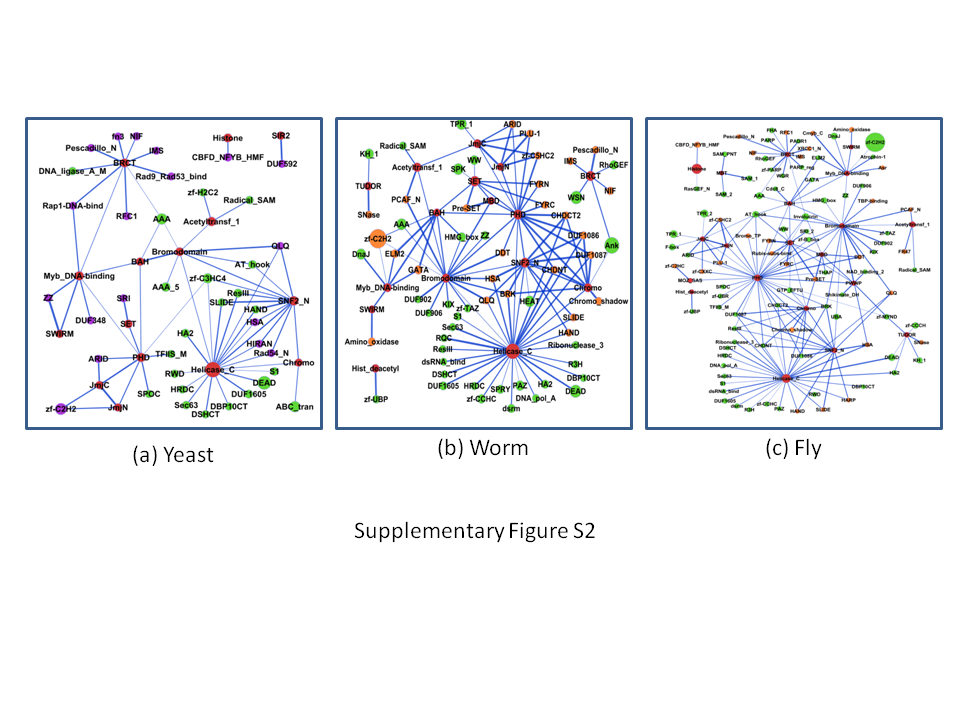

Supplement: Figure S2 — Domain co-occurrence network for known CM domains and their combination partners in yeast (a), worm (b), and fly (c). Nodes represent domains and each link represents co-occurrence relationship of two domains in proteins. Size of the nodes is proportional to domain abundance in each genome, and nodes are colored red, magenta and green, denoting known CM domains, candidate CM domains and non-CM domains, respectively. The thickness of edges is proportional to the Co-occurrence Score for the linked domain pair (See Materials and Methods for definition of Co-occurrence Score). (0.33 MB TIF) [file pone.0014122.s002.tif]

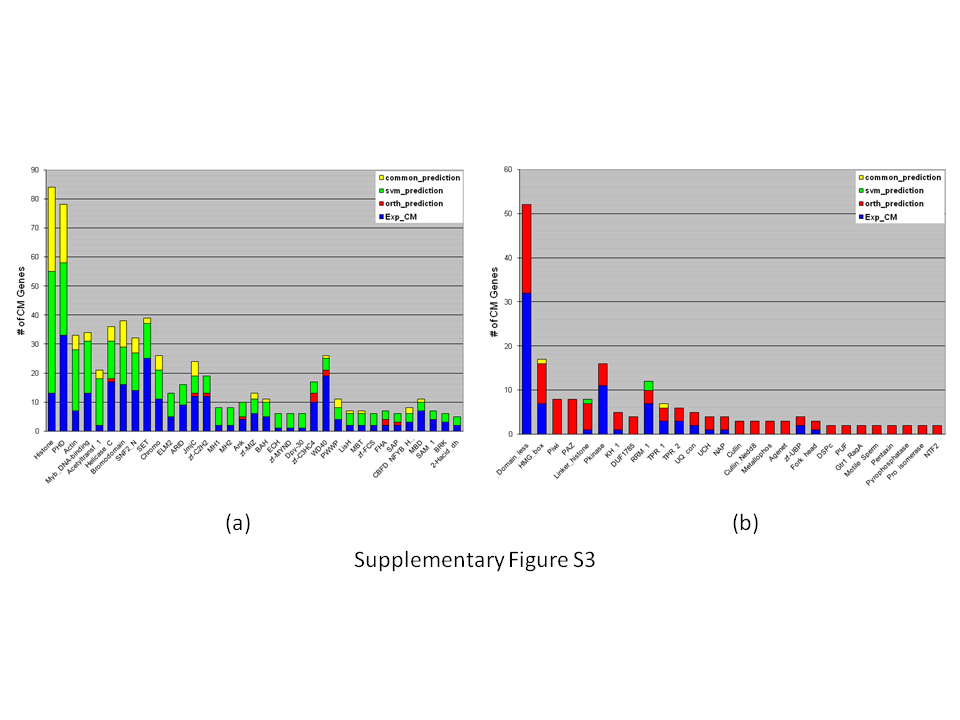

Supplement: Figure S3 — (a) Pfam domains that appear more frequently in our SVM predicted human CM genes than in those predicted by the orthology-based approach. Only the top 36 of 121 such domains are shown. (b) Pfam domains that appear more frequently in CM genes predicted by the orthology-based approach than in those predicted by our SVM-based approach approach. The top 28 of 60 such domains are shown. In both (a) and (b), “Exp_CM”: experimentally verified human CM genes (See SupplementaryTable 3). “svm_prediction”: CM genes predicted by our SVM-based approach only. “orth_prediction”: CM genes predicted by orthology-based approach only. “common_prediction”: CM genes predicted by both approaches. “_Domain_less” on the x-axis of panel (b) denotes CM genes that lack Pfam domain annotations. Note that the orthology-based approach is able to predict CM genes in the absence of Pfam domain annotations, while our SVM-based approach cannot. (0.19 MB TIF) [file pone.0014122.s003.tif]
